# Dr. Henry F. Blakeney

**Published:** 1897-05

**Authors:** 


### DR. HENRY F. BLAKENEY.

Died, at 143 Waverly Place, New York, March 13, 1897, Henry F. Blakeney, M.D., D.D.S., aged fifty-eight years.

Dr. Blakeney was a member of the first graduating class of the New York College of Dentistry, receiving his diploma December, 1869. He was also a graduate of the Medical Department of the University of Pennsylvania, but practised dentistry exclusively for twenty-eight years. He lays down his life a martyr from overwork.

